# Impact of COVID-19 on Perinatal Outcomes and Birth Locations in a Large US Metropolitan Area

**DOI:** 10.3390/healthcare12030340

**Published:** 2024-01-30

**Authors:** Esther G. Lee, Alejandra Perez, Arth Patel, Aloka L. Patel, Thaddeus Waters, Marielle Fricchione, Tricia J. Johnson

**Affiliations:** 1 Division of Neonatology, Department of Pediatrics, Rush University Medical Center, Chicago, IL 60612, USA; aloka_patel@rush.edu; 2 Department of Health Systems Management, Rush University, Chicago, IL 60612, USA; alejandra.perez@tuftsmedicine.org (A.P.); arth.patel@uchospitals.edu (A.P.); tricia_j_johnson@rush.edu (T.J.J.); 3 Department of Clinical Excellence, University of Chicago Medicine, Chicago, IL 60637, USA; 4 Department of Obstetrics & Gynecology, Rush University Medical Center, Chicago, IL 60612, USA; twaters3@buffalo.edu; 5 Department of Obstetrics & Gynecology, University at Buffalo, Buffalo, NY 14260, USA; 6 Division of Infectious Diseases, Department of Pediatrics, Rush University Medical Center, Chicago, IL 60612, USA; marielle_fricchione@rush.edu

**Keywords:** COVID-19, preterm birth, academic medical centers, birth hospital designation, length of stay

## Abstract

This was a population-based study to determine the impact of COVID-19 on birth outcomes in the Chicago metropolitan area, comparing pre-pandemic (April–September 2019) versus pandemic (April–September 2020) births. Multivariable regression models that adjusted for demographic and neighborhood characteristics were used to estimate the marginal effects of COVID-19 on intrauterine fetal demise (IUFD)/stillbirth, preterm birth, birth hospital designation, and maternal and infant hospital length of stay (LOS). There were no differences in IUFD/stillbirths or preterm births between eras. Commercially insured preterm and term infants were 4.8 percentage points (2.3, 7.4) and 3.4 percentage points (2.5, 4.2) more likely to be born in an academic medical center during the pandemic, while Medicaid-insured preterm and term infants were 3.6 percentage points less likely (−6.5, −0.7) and 1.8 percentage points less likely (−2.8, −0.9) to be born in an academic medical center compared to the pre-pandemic era. Infant LOS decreased from 2.4 to 2.2 days (−0.35, −0.20), maternal LOS for indicated PTBs decreased from 5.6 to 5.0 days (−0.94, −0.19), and term births decreased from 2.5 to 2.3 days (−0.21, −0.17). The pandemic had a significant effect on the location of births that may have exacerbated health inequities that continue into childhood.

## 1. Introduction

The COVID-19 pandemic widely impacted healthcare delivery, from individual decision-making to healthcare system operations. The care for pregnant mothers and subsequent neonatal outcomes during the pandemic has been a focus of interest throughout the world. Multiple population-based studies from Europe, South Korea, and Israel have demonstrated a reduction in the preterm birth (PTB) rate during the height of the pandemic and lockdown restrictions [1,2,3,4,5,6]; however, several studies found no difference in PTBs during the pandemic, including studies from Norway, Sweden, Denmark, and Canada [7,8,9]. Studies from the United States have also examined the effects of the pandemic on birth outcomes, although the majority have taken place in either a single hospital or hospital system [10,11,12,13] and included only a portion of hospitals from across the country [14,15,16] or included only commercially insured births [16], thereby limiting their generalizability to understand changes at the population level. Of the four population-based studies in the United States, all found a significant reduction in the PTB rate [17,18,19,20]; however, only one of these studies further investigated whether changes in PTB rates differed across socioeconomic characteristics such as race/ethnicity or payment source. Additionally, changes in day-to-day hospital operations and inadvertent changes to physical, social, and behavioral patterns of pregnant women in response to the pandemic, may have impacted the outcomes of deliveries and newborns in addition to the PTB rate, such as stillbirths, hospital location of delivery, or hospital length of stay (LOS). Furthermore, the impact of COVID-19 on these outcomes may have differed by socioeconomic status, further exacerbating infant and maternal disparities, however, few studies have examined differences beyond race and ethnicity such as source of payment.

There remains a paucity of data on how COVID-19 has affected healthcare delivery within the geographical bounds of a large metropolitan area, particularly in the United States. To elucidate the impact of the pandemic on labor and delivery and neonatal intensive care units (NICUs) in the Chicago metropolitan area, we investigated the rates of intrauterine fetal demise (IUFD) and stillbirths, PTBs, birth hospital designation (i.e., location of delivery), and maternal and infant hospital LOS preceding and during the pandemic. We hypothesized the following: (1) IUFD/stillbirth rates increased during the pandemic due to a reduction in access to face-to-face prenatal visits and maternal reluctance to seek hospital care when needed in fear of contracting COVID-19; (2) the PTB rate decreased during the pandemic due to both increases in IUFDs and stillbirths and a decrease in spontaneous PTBs; (3) the proportion of births that occurred in academic medical centers (AMCs) decreased during the pandemic due to AMCs being COVID-19-designated centers and a shift in non-COVID-19-related care to hospitals treating fewer patients with COVID-19; and (4) maternal and infant hospital LOS decreased during the pandemic to reduce exposure to COVID-19 and decrease non-COVID-19 inpatient census. As an exploratory aim, we examined whether changes in these outcomes differed by payment source, as a measure of socioeconomic status.

## 2. Materials and Methods

### 2.1. Study Design

This was a historical (retrospective) cohort study that included 90,931 births across eight counties (Cook, DuPage, Lake, Kane, Kankakee, Kendall, McHenry, and Will) in the Chicago metropolitan area in the state of Illinois, USA. Our risk factor of interest was exposure to the pandemic, with births classified as pre-COVID-19 pandemic (pre-pandemic) (1 April–30 September 2019) or during the COVID-19 pandemic (pandemic) (1 April–30 September 2020). We chose the periods of the study to start in April, because states across the United States began to implement shutdowns on 15 March 2020, and statewide stay-at-home orders were implemented on 21 March 2020. Therefore, we excluded births that occurred between January and March 2020 to ensure we were including births that occurred after the announcement and implementation of stay-at-home orders within the Chicago metropolitan area. Additionally, we limited the 2 historical cohorts to births of the same calendar months (April–September) to control for the potential effects of seasonality on birth outcomes. Because our pandemic cohort was limited to births in a 6-month period in 2020, we examined a matched pre-pandemic cohort in the calendar year of 2019. Additionally, complete data about neonatal hospitalization were only available after the infant’s discharge. Due to the lag in obtaining data for infants who had prolonged LOS, our access to data was limited to the 6-month periods before and during the pandemic.

### 2.2. Data Sources

We obtained the data from the Illinois Hospital Association’s COMPData data files, which provide data for benchmarking and comparative purposes. We obtained neighborhood-level socioeconomic characteristics from the US Census Bureau’s American Factfinder at the zip code level for the five-year average of 2015–2019 and linked these data to hospital discharge records based on the zip code of home residence [21]. We excluded records with missing data for any analytic variables from the analysis, including 1001 records with a home zip code that did not match a census tract, 127 records with missing neighborhood characteristics for a census tract, 2304 records with missing or unknown race/ethnicity, and 19 records with unknown gender. Of the 94,382 births in the data set, 90,931 (96.3%) records had complete information and were included in the analysis. Overall, the sample included 58 hospitals (5 AMCs), with the number of births during the study period ranging from 21 to 11,658. The median number of births per hospital was 1083 (interquartile range [IQR]: 658, 2201). 

### 2.3. Measures

Outcomes included IUFD/stillbirth, PTB (birth gestational age (GA) < 37 weeks), birth hospital designation (AMC (n = 5) or other hospital), and infant and maternal LOS. IUFD/stillbirths included maternal discharge record ICD10 diagnosis codes (ICD10) of O36.4XXx (IUFD) and Z37.1, Z37.3, Z37.4, Z37.6x, and Z37.7 (stillbirth). PTBs included ICD10 P07.xx. We calculated the maternal and infant hospital LOS by subtracting the respective admission date from the discharge date. Additionally, maternal indication for delivery was classified into spontaneous PTB (ICD10: O42 and O60), indicated PTB (ICD10: O11.x, O41, O43, O44, O45, and O46), unspecified PTB (not classified as either spontaneous or indicated), or term birth.

Demographic characteristics included race/ethnicity (Hispanic, non-Hispanic Asian [Asian], non-Hispanic Black [Black], non-Hispanic White [White], other racial groups) and primary payment source (commercial insurance, Medicaid/uninsured, other payers (Medicare, not specified insurance plans). Birth GA was further classified into <28 0/7 weeks, 28 0/7–31 6/7 weeks, 32 0/7–36 6/7 weeks, and ≥37 0/7 weeks based on ICD10. Other variables included whether the infant was transferred out from the birth hospital to another facility and infant mortality during the birth hospitalization. To assess concordance between infant acuity and birth hospital neonatal/perinatal level of care for very PTBs (GA < 32 weeks), we created a variable to indicate the appropriate or inappropriate initial level of neonatal care, as very preterm (VPT) infants born at a hospital without risk-appropriate care would necessitate a postnatal transfer to a higher-level NICU. VPT infants born at a hospital with a Level III NICU (the highest designation in Illinois) and infants with birth GAs between 30 0/7 and 31 6/7 weeks born at a hospital with either a Level II Extended or Level III NICU were classified as having appropriate care. VPT infants born at a hospital without these capabilities were classified as having inappropriate neonatal care. 

Neighborhood socioeconomic characteristics included percent of the population in the labor force (i.e., either working or unemployed and looking for work), percent of housing units that were considered crowded (1.01 or more occupants per room), percent of households without a computer, percent of adults age 25 and older with at least a high school degree, and whether the neighborhood was defined as having concentrated poverty (at least 20% of the population living below the federal poverty level) [21]. 

### 2.4. Statistical Analysis

We used frequency distributions to describe the sample characteristics. We compared unadjusted mean hospital LOS for preterm versus term births and by type of delivery (spontaneous preterm, indicated preterm, unspecified preterm, or term) using means and standard deviations. We used binary logistic regression models to estimate the likelihood of stillbirth/IUFD and PTB between pandemic eras and generalized linear regression models with a negative binomial distribution and log link function to estimate the relative rate of hospital LOS. All regression models controlled for race/ethnicity, primary payment source, infant sex, and neighborhood characteristics (percent of the population in the labor force, percent of housing units that were considered crowded, percent of households without a computer, percent of adults age 25 and older with at least a high school degree, and whether the neighborhood was defined as having concentrated poverty). Robust standard errors clustered at the zip code level were computed for each model. Additionally, separate LOS models were estimated for PTB versus term birth and by maternal indication for delivery (spontaneous PTB, indicated PTB, unspecified PTB, or term birth). For each model, the predictive margin and average marginal effect in days were calculated by first computing the predicted outcome, assuming all subjects were in the pre-pandemic and then the pandemic periods. The 95% confidence intervals were computed using the method of recycled predictions. We used Stata version 17.0 and SAS version 9.4 for all statistical analyses.

## 3. Results

The sample included 43,555 pandemic births (April–September 2020) and 47,376 pre-pandemic (April–September 2019), including 593 IUFD/stillbirth encounters (0.7%) (Table 1).

The unadjusted incidence of IUFD/stillbirths was 285 encounters (0.7%) during the pandemic versus 308 encounters (0.7%) in the pre-pandemic era (*p* = 0.937). The unadjusted incidence of PTBs was 9.1% during the pandemic versus 9.3% in the pre-pandemic era (*p* = 0.217). After adjusting for infant and neighborhood characteristics, there were no differences in the IUFD/stillbirth or the PTB rates between eras (Table 2). 

### 3.1. Maternal Delivery Indication and Outcomes

Overall, there were a total of 90,051 deliveries (Supplemental Table S1) and maternal indications for delivery were similar between eras (*p* = 0.052). After adjusting for maternal and neighborhood characteristics, there was no difference in the adjusted probability of medically indicated PTB (2.7% pandemic versus 2.8% pre-pandemic), spontaneous PTB (4.9% pandemic versus 5.0% pre-pandemic), unspecified PTB (1.7% pandemic versus 1.8% pre-pandemic), or term birth (90.7% pandemic versus 90.4% pre-pandemic) (Table 2).

### 3.2. Changes in Birth Location by Hospital Designation

**Figure 1:**
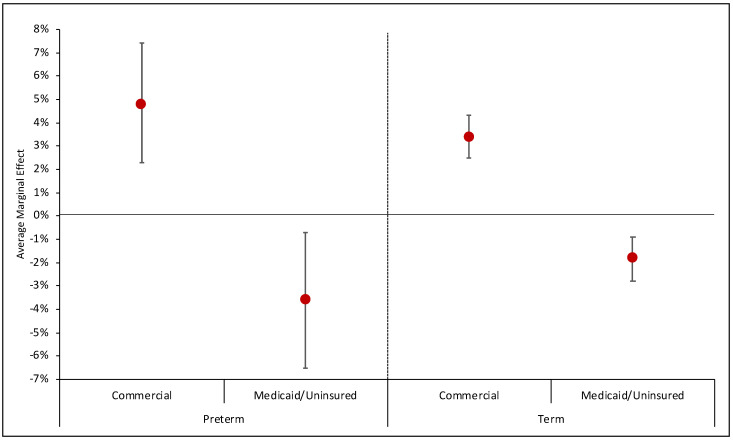
Average marginal effect of COVID-19 on probability of birth in an academic medical center by primary payer for preterm and term births. Note: Average Marginal Effect = Difference in probability of AMC birth for all infants in group as if they were born during COVID-19 and probability of AMC birth for all infants in group as if they were born before COVID-19.

Pandemic and pre-pandemic AMC births accounted for 22.6% and 21.2% of total births (*p* < 0.001). After adjusting for infant and neighborhood characteristics, the predicted probability of pandemic and pre-pandemic AMC birth was 22.4% (95% confidence interval [CI] 19.7, 25.1) and 21.4% (95% CI 18.8, 24.1), or an adjusted difference of 1.0 percentage point (95% CI 0.4, 1.5) (Table 2). We found no difference in birth hospital designation for PTBs overall. Pandemic and pre-pandemic AMC births were as follows: commercially insured PTBs 33.4% vs. 25.2% (adjusted difference: 4.8 percentage points, 95% CI 2.3, 7.4) and Medicaid/uninsured PTBs 23.4% vs. 29.2% (adjusted difference: −3.6 percentage points, 95% CI 6.5, −0.7) (Figure 1; Supplemental Table S2); for commercially insured term births, 29.0% vs. 24.0% (adjusted difference: 3.4 percentage points, 95% CI 2.5, 4.3); and Medicaid/uninsured term births 14% vs. 17.6% (adjusted difference: −1.8 percentage points, 95% CI −2.8, −0.9).

### 3.3. Hospital Length of Stay

**Figure 2:**
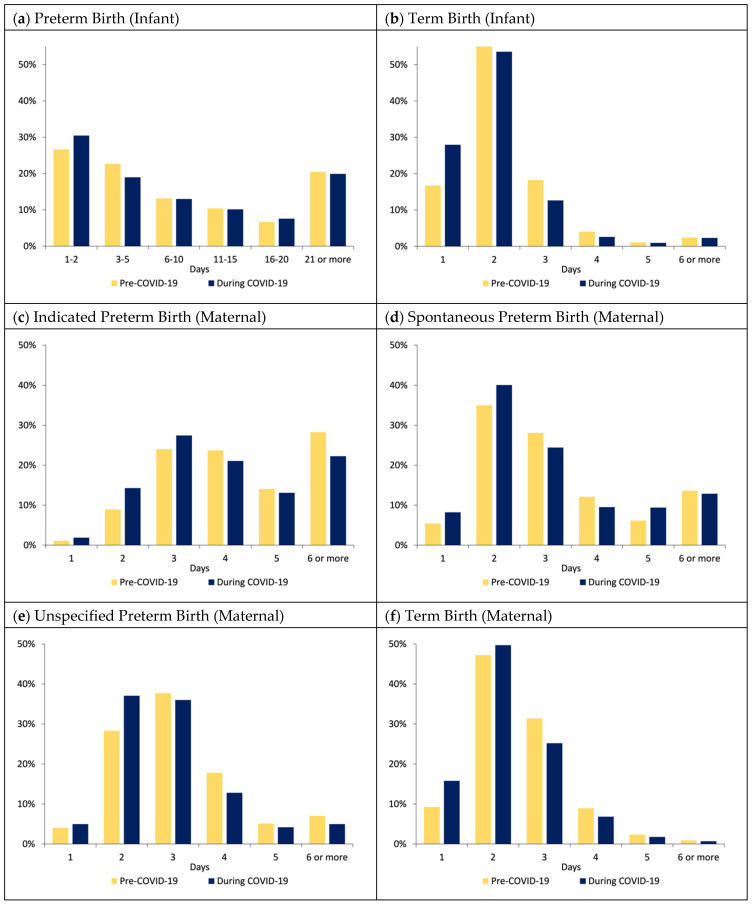
Infant and maternal hospital length of stay by COVID-19 era. (**a**) Preterm birth (infant); (**b**) term birth (infant); (**c**) indicated preterm birth (maternal); (**d**) spontaneous preterm birth (maternal); (**e**) unspecified preterm birth (maternal); (**f**) term birth (maternal).

**Figure 3:**
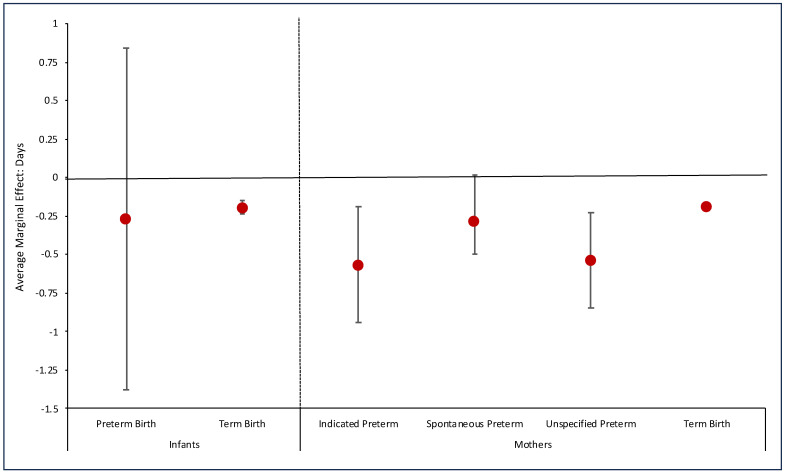
Average marginal effect of COVID-19 on probability of birth in an academic medical center by primary payer for preterm and term infant and maternal hospital length of stay. Note: Average Marginal Effect = Difference in hospital length of stay for all infants or mothers in group as if they were born during COVID-19 and hospital length of stay for all infants or mothers in group as if they were born before COVID-19.

Infant LOS decreased between the pre-pandemic and pandemic eras, decreasing from 3.6 days to 3.4 days (*p* < 0.001), with an adjusted difference of −0.27 days (95% CI −0.35, −0.20). There was no difference in hospital LOS for PTBs; however, term birth LOS decreased from 2.4 days to 2.2 days pre-pandemic to pandemic (adjusted difference: −0.20 days) (Figure 2 and Figure 3; Supplemental Table S3). Maternal LOS for indicated PTBs decreased pre-pandemic to pandemic 5.6 days to 5.0 days (adjusted difference: −0.57 days); maternal LOS for term births decreased from 2.5 days to 2.3 days (adjusted difference: −0.19 days), mirroring that of the decrease in term infant hospital LOS.

## 4. Discussion

PTB is the leading cause of mortality in children <5 years of age, with an estimated 15 million PTBs worldwide [22]. Speculations that pandemic lockdown-mandated physical, psychosocial, environmental, and occupational alterations potentially led to fewer PTBs were intriguing. Our findings conflict with findings on PTBs from many European studies [2,5,23,24,25] and several population-based studies in the United States. Notably, population-based studies from Tennessee and Colorado found significant reductions in PTBs during the pandemic, with adjusted odds ranging from 0.86 (95% CI 0.79–0.93) in Tennessee at the height of the stay-at-home orders (22 March–30 April 2020) to 0.92 (95% CI 0.89–0.96) in Colorado in the first 9 months of the pandemic (April–December 2020) [18,19]. In the Chicago metropolitan area, we found no difference in the PTB rate for a similar time period (April–September, 2019 and 2020). Even after restricting the time period to the height of stay-at-home orders in Chicago (April–May 2020), we did not find a significant difference in PTBs. One plausible explanation for these differences is that the pandemic, and specifically the stay-at-home restrictions, had a greater effect on population movement in more rural geographic areas, such as those in Tennessee and Colorado, compared to more densely populated metropolitan areas, such as Chicago. Additionally, the duration and conditions of lockdowns varied across geographical locations, and the time frame of the analysis could have potentially led to differing results. Moreover, the incidence of medically indicated and spontaneous PTBs did not change during the pandemic in metropolitan Chicago, similar to other US studies [7,11,12,13,19,26]. Future work should examine the effect of the pandemic across geographic regions of the United States and globally using the same methods and same time periods of study to address these conflicting findings and quantify the effect of different mitigation strategies on infant and maternal outcomes.

We hypothesized IUFDs and stillbirths to have increased during the pandemic due to psychosocial factors, avoidance of care-seeking due to fear of contracting COVID-19 during pregnancy, a shift to telehealth appointments, and other obstetric practice changes. Although none of the population-based studies in the US included IUFD and stillbirths, our results are similar to other population-based studies from Sweden, Canada, France, and Denmark that found no significant difference in IUFDs and stillbirths during the pandemic [4,8,9,27]. Although studies from Australia, the United Kingdom, and Italy found increases in the IUFD and stillbirth rates [23,28,29], two of these studies were limited by including only a portion of births in the geographic region [23,29]. 

### 4.1. Shifts in Birth Location by Hospital Designation

We found a significantly higher proportion of overall births occurred at the five AMCs in Chicago during the height of the pandemic. Contrary to our hypothesis, this occurred despite the massive disruptions to hospital systems and scarcity of resources associated with the pandemic. Notably, we found a large increase in commercially insured term infants born at AMCs, and these non-complicated term births may not have necessitated the higher level of care at already overwhelmed AMCs. Future research should investigate the impact this shift had on hospital operations at the five AMCs in Chicago and if the change in birth location persisted beyond our study period. This finding demonstrates the importance of examining the effect of the pandemic on specific infant populations, given that the modest shift of birth location to AMCs overall masked important differences between commercially insured and Medicaid-insured infants. These findings can be utilized to guide public health systems in optimally allocating and preserving AMC beds for higher-risk term births and PTBs. 

### 4.2. Inequity

We found dramatic differences in AMC births by payment source during the pandemic that warrant future investigation. While the proportion of commercially insured PTBs at AMCs substantially increased during the pandemic, the proportion of PTBs with Medicaid/uninsured at AMCs simultaneously decreased. These changes are concerning, particularly in light of national disparities in access to high-quality care for high-risk mothers and newborns driven by sociodemographic factors [30,31].

Although we did not find significant differences in AMC births by race or ethnicity, our sample of infants with Medicaid/uninsured were predominantly non-White (Hispanic 29.6%, Black 24.4%, and White 21.4%) with the limitation of having 24.6% classified as Other. On the contrary, White infants represented the majority of those with commercial insurance (53%), with significantly lower proportions of Hispanic (12.7%) or Black infants (7.0%). Disparities in perinatal outcomes by race and ethnicity in the US are well documented, and non-Hispanic Black women are reported to be at a two-fold greater risk for preterm birth compared to non-Hispanic White women [32]. Furthermore, in a systematic review and meta-analysis of 4.3 million patients from 68 studies, socioeconomic disparity and clinical care quality were associated with mortality in racial and ethnic minority groups during COVID-19 [33]. Thus, shifts in AMC deliveries by payment source during the pandemic may have exacerbated existing disparities in maternal and infant access to healthcare related to social disadvantage, which is linked to racial/ethnic minority status in the US.

Another consideration was that mothers with Medicaid/uninsured, representing those with fewer economic resources, may have delivered at a lower-than-appropriate level facility, potentially leading to increased morbidity for their infants. It is uncertain whether the decrease in AMC births was driven by parent or obstetrician preference or if it reflected discordant care for the mothers and neonates. However, we reassuringly found no change in the proportion of VPT infants born at a hospital with inappropriate neonatal care, and there was no change in the postnatal transfers of infants between pandemic eras. Furthermore, our data suggest that there was no increased risk associated with postnatal neonatal transport to a higher-level NICU during the pandemic. This lack of demonstrated morbidity is reassuring; however, the shift in birth location to non-AMC hospitals with lower economic resources could have consequences for perinatal care and widen disparities. 

### 4.3. Hospital Length of Stay

We found relatively large reductions in LOS for mothers with indicated and unspecified PTBs, translating into more than a half-day shorter LOS. Additionally, both term infants and mothers delivering term infants had approximately 0.2-day shorter stays during the pandemic. This LOS reduction is remarkably similar to Molina et al.’s findings using data from 463 hospital members of the PINC AI Healthcare Database in the United States [14]. They demonstrated LOS decreased from 2.66 to 2.47 days on average, compared to 2.5 to 2.3 days for maternal term deliveries in our Chicago metropolitan sample. This reduction may be the direct result of adaptations in discharge protocols to reduce the transmission of the virus and reduce the burden on hospital capacity and operations or may be the byproduct of inadvertent change to expedite the discharge process. This aligns with reports of decreased LOS specifically in low-risk and controlled delivery settings [34,35,36]. Few studies reported negative feelings and experiences of parents in the hospital setting during the pandemic, which could have affected LOS and expedited the discharge process, such as women not feeling safe giving birth, parents reporting themes of loneliness, emotional distress, adverse breastfeeding experiences, and unanticipated changes in hospital policies [37,38,39]. 

It is important to note that these reductions in infant LOS could have consequences for the ability to detect neonatal complications such as jaundice, poor breastfeeding, dehydration, and infection during birth hospitalization. A Cochrane review of early discharge in term infants (defined variably as <24 h to <48 h after birth) versus standard discharge (defined most commonly as ≥48 h) demonstrated a moderate association between early discharge and infant readmission within the first 28 days of life [40]. While we found a 0.2-day difference in infant LOS, Figure 2 highlights the difference between eras in the proportion of term infants discharged by 1 or 2 days of life. Research studies examining the downstream effects of overall delivery changes and LOS during the pandemic are emerging and may shed light on the effects of these experiences. The findings may help guide hospital systems to balance the financial implications of earlier newborn discharges and potential readmissions while ensuring safety in outcomes. 

### 4.4. Limitations

Our study has notable limitations. First, we did not have access to out-of-hospital births and, nationally, home births increased by 22% between 2019 to 2022 [41]. However, home births represent only 0.5% of total births in Illinois and are unlikely to account for the changes observed in our study [42]. Second, our neighborhood characteristics were measured at the zip code level and may not have been sufficiently granular to capture the variation in community characteristics. Third, the birth and delivery records are independent of one another, so there may be a discrepancy between the number of births and the number of deliveries. Fourth, we did not have access to readmission data to assess downstream medical risks due to LOS reductions. Although data from one New York hospital system suggest that the LOS reduction was not offset by an increase in maternal readmissions in the 30 days after delivery, more research is needed to examine the longer-term consequences and safety of these pandemic-induced birth changes [35]. Fifth, we focused on the PTB type of indicated vs. spontaneous with the hypothesis that the pandemic affected many aspects of prenatal care delivery. However, we did not examine if this led to differences in the mode of delivery (i.e., Cesarean section vs. vaginal births). Finally, we did not have access to information about hospital-specific protocols implemented during the pandemic that could have facilitated earlier hospital discharge for both mothers and infants.

## 5. Conclusions

In our representative sample of Illinois hospitals in the eight Chicago metropolitan counties, we did not demonstrate a change in PTBs, IUFDs, and stillbirths. We did, however, find correlations between primary payment source and birth hospital designation with birth outcomes that represent a potential driver of healthcare disparities. Further studies are needed to examine the downstream implications, if any, on health outcomes of mothers and infants, and to explore if this correlation persists after the conclusion of the pandemic health emergency. Finally, it is important to study the financial and operational impacts that may have resulted from these delivery alterations. These data from a large population cohort of over 90,000 births provide critical information that may inform healthcare systems as they encounter future pandemics.

## Figures and Tables

**Table 1:** Description of the sample of liveborn infants.

	Pre-COVID-19 April–September 2019 N = 47,068	During COVID-19 April–September 2020 N = 43,270	*p*-Value
Male, n (%)	24,212 (51.4)	22,165 (51.2)	0.517
Primary payer, n (%)			<0.001
Commercial	26,888 (57.1)	23,516 (54.4)	
Medicaid/uninsured	19,803 (42.1)	19,476 (45.0)	
Other	377 (0.8)	278 (0.6)	
Race/ethnicity, n (%)			<0.001
Asian	2772 (5.9)	2657 (6.1)	
Black	7062 (14.9)	6329 (14.5)	
Hispanic	9147 (19.3)	9115 (20.9)	
White	17,907 (37.8)	17,698 (40.6)	
Other	10,488 (22.1)	7756 (17.8)	
Gestational age, n (%)			0.263
<28 weeks	270 (0.6)	215 (0.5)	
28–<32 weeks	352 (0.8)	333 (0.8)	
32–<37 weeks	3758 (8.0)	3365 (7.8)	
37 weeks or older	42,679 (90.7)	39,338 (91.0)	
<32 weeks gestational age born at hospital with inappropriate level of NICU care, n (%)	62/622 (10.0)	57/548 (10.4)	0.807
All infants transferred to another facility, n (%)	479 (1.0)	388 (0.9)	0.062
In-hospital death, n (%)	125 (0.3)	103 (0.2)	0.410
Neighborhood characteristics			
Concentrated poverty, >20% below federal poverty level, n (%)	7272 (15.5)	6696 (15.5)	0.918
% population working, mean (sd)	67.3 (6.6)	67.4 (6.6)	0.052
% housing units crowded (more than 1 person per room), mean (sd)	3.2 (2.4)	3.2 (2.4)	0.042
% households without a computer, mean (sd)	9.4 (5.6)	9.3 (5.5)	0.054
% population age 25 and older with high school degree or more, mean (sd)	87.3 (9.3)	87.4 (9.3)	0.139

Chi-square tests used to examine association between each categorical variable and pandemic era, and independent samples *t*-tests used to examine association between each continuous variable and pandemic era.

**Table 2:** Logistic regression results for infant and maternal outcomes.

		N (%)			Adjusted				
	N	Pre-COVID-19 n (%)	During COVID-19 n (%)	*p*-Value *	OR (95% CI)	*p*-Value **	Marginal Effect		
							Predictive Margin		Average Marginal Effect (%) (95% CI)
							Pre-COVID-19 Prob (95% CI)	During COVID-19 Prob (95% CI)	
Stillbirth/IUFD	90,931	308 (0.7)	285 (0.7)	0.937	1.00 (0.85, 1.18)	0.971	0.7 (0.6, 0.7)	0.7 (0.6, 0.7)	0.0 (−0.1 to 0.1)
Preterm Birth	90,338	4389 (9.3)	3932 (9.1)	0.217	0.98 (0.94, 1.03)	0.442	9.3 (9.0, 9.6)	9.1 (8.8, 9.4)	−0.1 (−0.5, 0.2)
Birth Category	90,051			0.502					
Indicated Preterm	2465	1288 (2.8)	1177 (2.7)		0.99 (0.92, 1.07)	0.761	2.7 (2.6, 2.9)	2.7 (2.6, 2.9)	−0.0 (−0.2 to 0.2)
Spontaneous Preterm	4445	2341 (5.0)	2104 (4.9)		0.97 (0.91, 1.03)	0.268	5.0 (4.8, 5.2)	4.9 (4.7, 5.1)	−0.2 (−0.4 to 0.1)
Unspecified Preterm	1583	841 (1.8)	742 (1.7)		0.95 (0.86, 1.04)	0.277	1.8 (1.7, 1.9)	1.7 (1.6, 1.8)	−0.1 (−0.3 to 0.1)
Term	81,558	42,259 (90.4)	39,299 (90.7)		REF		90.4 (90.2, 90.7)	90.7 (90.5, 91.0)	−0.2 (−0.7 to 0.6)
AMC Birth Location	90,338	9997 (21.2)	9791 (22.6)	<0.001	1.07 (1.03, 1.12)	0.002	21.4 (18.8, 24.1)	22.4 (19.7, 25.1)	1.0 (0.4, 1.5)

* *p*-value based from Chi-square test; ** *p*-value from binary logistic regression, controlling for race/ethnicity, primary payment source, infant sex, and neighborhood characteristics (percent of the population in the labor force, percent of housing units that were considered crowded, percent of households without a computer, percent of adults age 25 and older with at least a high school degree, and whether the neighborhood was defined as having concentrated poverty).

## Data Availability

Reasonable requests can be submitted to the corresponding author.

## Supplementary Materials

The following supporting information can be downloaded at: https://www.mdpi.com/article/10.3390/healthcare12030340/s1, Table S1. Description of mothers, N = 90,051; Table S2. Generalized linear regression results for birth at academic medical center versus community hospitals before versus during COVID-19 by primary payer; Table S3. Generalized linear regression results for infant and maternal length of hospital stay.
