# Supplementary material for: Impact of COVID-19 on Perinatal Outcomes and Birth Locations in a Large US Metropolitan Area
Source: Healthcare (Basel). 2024 Jan 30;12(3):340. doi: 10.3390/healthcare12030340 (PMC10855483; doi:10.3390/healthcare12030340)
Supplement: Supplementary file 1 [file healthcare-12-00340-s001.zip › healthcare-2810113-supplementary.pdf]

Supplemental Table S1. Description of Mothers, N = 90,051.

	Pre-COVID-19 Apr – Sept 2019 N = 46,729	During COVID-19 Apr – Sept 2020 N = 43,322	p-value
Race/ethnicity, n (%)			<0.001
Asian	3071 (6.6)	2809 (6.5)	
Black	7884 (16.9)	7203 (16.6)	
Hispanic	10,769 (23.1)	10,388 (24.0)	
White	20,396 (43.7)	18,959 (43.8)	
Other	4609 (9.9)	3963 (9.2)	
Primary payer, n (%)			<0.001
Commercial	28,028 (60.0)	24,694 (57.0)	
Medicaid/Uninsured	18,172 (38.9)	18,227 (42.1)	
Other	529 (1.1)	401 (0.9)	
Transferred to another hospital, n (%)	24 (0.05)	17 (0.04)	0.394

Supplemental Table S2. Generalized Linear Regression Results for Birth at Academic Medical Center versus Community Hospitals Before versus During COVID-19 by Primary Payer.

		AMC Birth N (%)			Adjusted Model				
							Marginal Effect		
							Predictive Margin		
	N	Pre-COVID-19	During COVID-19	p-value	Adjusted OR (95% CI)	p-value	Pre-COVID-19 % (95% CI)	During COVID-19 % (95% CI)	Average Marginal Effect (%)
Preterm Birth	8321	1168 (26.6)	1130 (28.8)	0.030	1.04 (0.93, 1.17)	0.461	27.3 (24.2, 30.3)	28.0 (24.7, 31.2)	0.7 (-1.2 to 2.5)
Commercial	4190	575 (25.2)	675 (33.4)	<0.001	1.36 (1.16, 1.59)	<0.001	26.5 (23.0, 30.0)	31.3 (27.4, 35.2)	4.8 (2.3 to 7.4)
Medicaid/ Uninsured	4150	627 (29.2)	470 (23.4)	<0.001	0.80 (0.66, 0.96)	0.014	28.3 (24.9, 31.8)	24.7 (21.4, 28.1)	-3.6 (-6.5 to -0.7)
Term Birth	82,017	8829 (20.7)	8661 (22.0)	<0.001	1.05 (1.01, 1.10)	0.021	21.0 (18.2, 23.7)	21.7 (18.9, 24.5)	0.7 (0.1 to 1.4)
Commercial	46,214	6085 (24.0)	6480 (29.0)	<0.001	1.25 (1.18, 1.32)	<0.001	24.0 (20.7, 27.4)	27.4 (23.9, 31.0)	3.4 (2.5 to 4.3)
Medicaid/ Uninsured	35,210	3138 (17.6)	2538 (14.0)	<0.001	0.85 (0.78, 0.91)	<0.001	16.8 (14.4, 19.2)	15.0 (12.8, 17.2)	-1.8 (-2.8 to -0.9)

Supplemental Table S3. Generalized Linear Regression Results for Infant and Maternal Length of Hospital Stay.

		Unadjusted Means (sd)			Adjusted Model				
							Marginal Effect		
							Predictive Margin, Length of Stay		
	N	Pre-COVID-19 Mean (sd)	During COVID-19 Mean (sd)	p-value	Adjusted RR (95% CI)	p-value	Pre-COVID-19 Days (95% CI)	During COVID-19 Days (95% CI)	Average Marginal Effect (days)
Infant Length of Stay	90,338	3.6 (9.0)	3.4 (8.8)	<0.001	0.93 (0.91, 0.94)	<0.001	3.6 (3.6, 3.7)	3.4 (3.3, 3.4)	-0.27 (-0.34 to -0.20)
Preterm Birth	8321	15.8 (24.1)	15.6 (24.3)	0.695	0.98 (0.92, 1.05)	0.630	15.8 (15.0, 16.6)	15.5 (14.8, 16.2)	-0.27 (-1.38 to 0.84)
Term Birth	82,017	2.4 (3.7)	2.2 (3.0)	<0.001	0.92 (0.90, 0.93)	<0.001	2.4 (2.3, 2.4)	2.2 (2.1, 2.2)	-0.20 (-0.24 to -0.15)
Maternal Length of Stay									
Indicated Preterm	2465	5.6 (5.3)	5.0 (4.7)	0.005	0.90 (0.84, 0.96)	0.003	5.6 (5.3, 5.9)	5.0 (4.7, 5.3)	-0.57 (-0.94 to -0.19)
Spontaneous Preterm	4445	4.1 (4.9)	3.9 (5.2)	0.116	0.94 (0.88, 1.00)	0.065	4.1 (3.9, 4.3)	3.9 (3.7, 4.1)	-0.42 (-0.50 to 0.02)
Unspecified Preterm	1583	3.7 (4.6)	3.1 (2.5)	0.002	0.85 (0.78, 0.93)	0.001	3.7 (3.4, 4.0)	3.2 (3.0, 3.3)	-0.54 (-0.85 to -0.23)
Term	81,558	2.5 (1.0)	2.3 (1.0)	<0.001	0.92 (0.92, 0.93)	<0.001	2.5 (2.5, 2.5)	2.3 (2.3, 2.3)	-0.19 (-0.21 to -0.17)
